# COVID-19 Vaccination Coverage and Demographic Characteristics of Infants and Children Aged 6 Months–4 Years — United States, June 20–December 31, 2022

**DOI:** 10.15585/mmwr.mm7207a4

**Published:** 2023-02-17

**Authors:** Bhavini Patel Murthy, Hannah E. Fast, Elizabeth Zell, Neil Murthy, Lu Meng, Lauren Shaw, Tara Vogt, Kevin Chatham-Stephens, Tammy A. Santibanez, Lynn Gibbs-Scharf, LaTreace Q. Harris

**Affiliations:** ^1^Immunization Services Division, National Center for Immunization and Respiratory Diseases, CDC; ^2^CDC COVID-19 Emergency Response Team; ^3^Stat-Epi Associates, Inc., Ponte Vedra Beach, Florida; ^4^Division of Human Development and Disability, National Center for Birth Defects and Developmental Disabilities, CDC.

Although severe COVID-19 illness and hospitalization are more common among older adults, children can also be affected (*1*). More than 3 million cases of COVID-19 had been reported among infants and children aged <5 years (children) as of December 2, 2022 (*2*). One in four children hospitalized with COVID-19 required intensive care; 21.2% of cases of COVID-19–related multisystem inflammatory syndrome in children (MIS-C) occurred among children aged 1–4 years, and 3.2% of MIS-C cases occurred among infants aged <1 year (*1*,*3*). On June 17, 2022, the Food and Drug Administration issued an Emergency Use Authorization (EUA) of the Moderna COVID-19 vaccine for children aged 6 months–5 years and the Pfizer-BioNTech COVID-19 vaccine for children aged 6 months–4 years. To assess COVID-19 vaccination coverage among children aged 6 months–4 years in the United States, coverage with ≥1 dose* and completion of the 2-dose or 3-dose primary vaccination series^†^ were assessed using vaccine administration data for the 50 U.S. states and District of Columbia submitted from June 20 (after COVID-19 vaccine was first authorized for this age group) through December 31, 2022. As of December 31, 2022, ≥1-dose COVID-19 vaccination coverage among children aged 6 months–4 years was 10.1% and was 5.1% for series completion. Coverage with ≥1 dose varied by jurisdiction (range = 2.1% [Mississippi] to 36.1% [District of Columbia]) as did coverage with a completed series (range = 0.7% [Mississippi] to 21.4% [District of Columbia]), respectively. By age group, 9.7 % of children aged 6–23 months and 10.2% of children aged 2–4 years received ≥1 dose; 4.5% of children aged 6–23 months and 5.4% of children aged 2–4 years completed the vaccination series. Among children aged 6 months–4 years, ≥1-dose COVID-19 vaccination coverage was lower in rural counties (3.4%) than in urban counties (10.5%). Among children aged 6 months–4 years who received at least the first dose, only 7.0% were non-Hispanic Black or African American (Black), and 19.9% were Hispanic or Latino (Hispanic), although these demographic groups constitute 13.9% and 25.9% of the population, respectively (*4*). COVID-19 vaccination coverage among children aged 6 months–4 years is substantially lower than that among older children (*5*). Efforts are needed to improve vaccination coverage among children aged 6 months–4 years to reduce COVID-19–associated morbidity and mortality.

Data on COVID-19 vaccine administration in the United States are reported to CDC by jurisdictions, pharmacies, and federal entities through immunization information systems (IISs),^§^ the Vaccine Administration Management System (VAMS),^¶^ or through direct data submission.** Children aged 6 months–4 years residing in one of 50 states or the District of Columbia who received ≥1 COVID-19 vaccine dose as of December 31, 2022, and whose data were reported to CDC by February 9, 2023, were included in this analysis.^††^

Daily and cumulative numbers of children initiating COVID-19 vaccination were calculated. Receipt of ≥1 COVID-19 vaccine dose and series completion among children aged 6 months–4 years were calculated overall and by age group^§§^ (6–23 months and 2–4 years), sex (male and female), and jurisdiction (50 states and the District of Columbia). Population size by age group and sex were obtained for the 50 states and District of Columbia from the U.S. Census Bureau’s 2020 Population Estimates Program (*4*). Vaccination coverage with the first dose and series completion was calculated. Tests for statistical significance were not conducted because these data reflect the U.S. population and were not based on population samples.

Race and ethnicity data were available for 71.4% of children aged 6 months–4 years and were analyzed by the following categories: Black, Hispanic, non-Hispanic American Indian or Alaska Native, non-Hispanic Asian (Asian), non-Hispanic Native Hawaiian or other Pacific Islander, non-Hispanic White (White), and non-Hispanic multiple races or other (multiracial/other). The percentage of children aged 6 months–4 years receiving the first dose of COVID-19 vaccine was calculated by race and ethnicity.

To investigate disparities in vaccination coverage by urban-rural environment, first-dose coverage was also calculated by two- and six-level urban-rural classifications according to the 2013 National Center for Health Statistics (NCHS) urban-rural classification scheme (*6*). To dichotomize counties as urban versus rural, four of these six categories (large central metropolitan, large fringe metropolitan, medium metropolitan, and small metropolitan) were combined and considered urban areas, and two (micropolitan and noncore) were combined and considered as rural areas (*6*). Eight counties in California with <20,000 residents were excluded from the analysis because of data-sharing restrictions on county-level information reported to CDC. All analyses were conducted using SAS software (version 9.4; SAS Institute). This activity was reviewed by CDC and was conducted consistent with applicable federal law and CDC policy.^¶¶^

As of December 31, 2022, a total of 1,755,596 (10.1%) children aged 6 months–4 years had received ≥1 dose of a COVID-19 vaccine (Table), and approximately 39% of these children received the first dose within 1 month of vaccine authorization (Supplementary Figure, https://stacks.cdc.gov/view/cdc/124660). Overall, 5.1% of children in this age group completed the series during the study period (Supplementary Table, https://stacks.cdc.gov/view/cdc/124661). Among those who received their first dose of Pfizer-BioNTech vaccine by September 4, 2022, or of Moderna vaccine by October 23, 2022, approximately 70% had completed the vaccination series.*** COVID-19 vaccination coverage with ≥1 dose varied by jurisdiction (range = 2.1% [Mississippi] to 36.1% [District of Columbia]), as it did for series completion (0.7% [Mississippi] to 21.4% [District of Columbia]), with lower coverage in the southeastern United States (Figure 1). Coverage was slightly higher among children aged 2–4 years (10.2% for ≥1 dose; 5.4% for series completion) than among those aged 6–23 months (9.7% for ≥1 dose; 4.5% for series completion). Coverage was similar among males and females.

**TABLE:** Vaccination coverage among children aged 6 months–4 years who received ≥1 dose* of a COVID-19 vaccination series, by jurisdiction,^†^ sex,^§^ age group,^¶^ and urban-rural classification** — United States, June 20–December 31, 2022

Jurisdiction	No. vaccinated (%)												
	Total	Sex		Age group		Urban-rural classification							
						Two-level		Six-level					
		Female	Male	6–23 mos	2–4 yrs	Urban	Rural	Large metropolitan	Large fringe metropolitan	Medium metropolitan	Small metropolitan	Micropolitan	Noncore
**United States**	**1,755,596 (10.1)**	**858,987 (10.1)**	**889,394 (10.0)**	**547,089 (9.7)**	**1,208,507 (10.2)**	**1,587,826 (10.5)**	**79,527 (3.4)**	**693,265 (12.5)**	**487,550 (11.3)**	**310,526 (8.4)**	**96,485 (6.2)**	**53,737 (3.8)**	**25,790 (2.7)**
Alabama	**8,122 (3.1)**	3,998 (3.1)	4,122 (3.1)	2,621 (3.1)	5,501 (3.1)	7,249 (3.5)	625 (1.1)	2,694 (7.2)	520 (2.0)	2,646 (3.6)	1,389 (2.1)	380 (1.3)	245 (0.8)
Alaska	**4,141 (9.2)**	2,017 (9.3)	2,109 (9.1)	1,420 (9.8)	2,721 (8.9)	3,034 (10.0)	1,081 (7.5)	—	—	2,651 (10.9)	383 (6.3)	304 (14.0)	777 (6.3)
Arizona	**34,356 (8.9)**	16,892 (9.0)	17,438 (8.9)	10,081 (8.1)	24,275 (9.3)	30,561 (8.4)	3,369 (17.0)	16,847 (6.8)	1,006 (4.3)	8,173 (16.2)	4,535 (10.5)	2,168 (15.2)	1,201 (21.5)
Arkansas	**7,254 (4.3)**	3,496 (4.3)	3,636 (4.2)	2,228 (4.1)	5,026 (4.4)	6,367 (5.8)	757 (1.3)	—	23 (0.7)	5,929 (6.9)	415 (2.0)	437 (1.5)	320 (1.1)
California	**267,893 (12.8)**	131,621 (12.9)	136,161 (12.8)	84,860 (12.6)	183,033 (12.9)	264,101 (12.9)	1,937 (5.0)	189,611 (14.8)	43,291 (15.4)	26,119 (6.2)	5,080 (7.3)	1,752 (6.7)	185 (1.5)
Colorado	**51,075 (17.4)**	25,189 (17.5)	25,868 (17.3)	18,076 (19.1)	32,999 (16.6)	48,183 (18.5)	2,634 (8.1)	11,758 (31.7)	23,615 (20.4)	12,072 (13.2)	738 (4.6)	1,771 (9.5)	863 (6.2)
Connecticut	**25,415 (15.6)**	12,449 (15.7)	12,956 (15.6)	7,337 (14.1)	18,078 (16.4)	24,396 (15.7)	838 (12.5)	6,862 (16.0)	2,158 (19.0)	15,376 (15.1)	—	838 (12.5)	—
Delaware	**4,865 (9.9)**	2,392 (9.9)	2,465 (9.8)	1,505 (9.5)	3,360 (10.1)	4,846 (9.8)	—	—	3,735 (13.2)	730 (6.8)	381 (3.8)	—	—
District of Columbia	**14,308 (36.1)**	6,964 (36.2)	7,309 (35.8)	5,445 (40.3)	8,863 (33.9)	14,187 (35.7)	—	14,187 (35.7)	—	—	—	—	—
Florida	**37,146 (3.6)**	18,521 (3.7)	18,592 (3.6)	10,298 (3.1)	26,848 (3.9)	36,578 (3.7)	266 (0.8)	16,812 (4.3)	10,900 (3.7)	8,223 (3.1)	643 (1.4)	165 (0.9)	101 (0.6)
Georgia	**32,055 (5.5)**	15,593 (5.4)	16,356 (5.5)	10,051 (5.3)	22,004 (5.6)	23,907 (4.9)	1,038 (1.1)	5,943 (11.1)	14,457 (5.2)	1,699 (2.5)	1,808 (2.0)	696 (1.2)	342 (0.9)
Hawaii	**8,616 (11.3)**	4,227 (11.5)	4,379 (11.2)	2,738 (11.2)	5,878 (11.4)	7,770 (12.5)	650 (4.7)	—	—	7,269 (13.6)	501 (5.8)	650 (4.7)	—
Idaho	**5,347 (5.2)**	2,561 (5.1)	2,786 (5.3)	1,688 (5.2)	3,659 (5.2)	4,367 (6.3)	931 (2.7)	—	—	3,617 (8.9)	750 (2.6)	758 (2.8)	173 (2.4)
Illinois	**84,131 (12.8)**	41,332 (12.8)	42,754 (12.7)	24,777 (11.7)	59,354 (13.3)	78,905 (13.4)	1,635 (2.3)	44,817 (16.6)	26,266 (12.3)	2,736 (5.6)	5,086 (9.5)	1,122 (2.7)	513 (1.8)
Indiana	**24,119 (6.4)**	11,728 (6.4)	12,364 (6.4)	8,117 (6.7)	16,002 (6.3)	22,338 (7.6)	1,535 (1.9)	5,679 (9.2)	9,570 (8.3)	2,901 (5.1)	4,188 (6.9)	1,117 (2.0)	418 (1.6)
Iowa	**17,473 (10.0)**	8,627 (10.1)	8,840 (10.0)	5,869 (10.5)	11,604 (9.8)	14,660 (13.7)	2,523 (3.8)	—	—	9,300 (12.9)	5,360 (15.2)	1,167 (4.5)	1,356 (3.3)
Kansas	**17,680 (10.7)**	8,693 (10.8)	8,975 (10.6)	5,863 (11.0)	11,817 (10.5)	15,501 (13.7)	1,320 (2.5)	—	11,018 (21.1)	1,643 (4.3)	2,840 (12.3)	922 (2.9)	398 (1.9)
Kentucky	**14,182 (5.8)**	6,932 (5.8)	7,241 (5.8)	4,557 (5.7)	9,625 (5.9)	11,861 (8.1)	2,066 (2.1)	5,080 (11.9)	2,792 (7.4)	3,203 (7.9)	786 (3.1)	1,178 (2.5)	888 (1.7)
Louisiana	**6,961 (2.6)**	3,409 (2.6)	3,548 (2.6)	1,978 (2.3)	4,983 (2.8)	6,472 (2.9)	450 (1.1)	2,171 (11.0)	1,597 (3.2)	2,069 (2.0)	635 (1.2)	350 (1.5)	100 (0.5)
Maine	**11,068 (19.3)**	5,400 (19.3)	5,654 (19.3)	3,831 (20.4)	7,237 (18.8)	8,477 (24.4)	2,421 (10.8)	—	—	7,267 (31.7)	1,210 (10.2)	778 (14.3)	1,643 (9.6)
Maryland	**57,217 (17.7)**	27,798 (17.5)	28,810 (17.5)	18,306 (17.3)	38,911 (17.8)	56,682 (17.9)	276 (4.0)	4,894 (15.4)	50,461 (19.4)	696 (4.3)	631 (6.8)	167 (5.3)	109 (2.9)
Massachusetts	**68,704 (21.6)**	33,917 (21.9)	34,772 (21.4)	17,055 (16.5)	51,649 (24.1)	64,375 (20.5)	506 (12.8)	8,353 (23.3)	45,874 (23.0)	9,252 (13.9)	896 (7.8)	505 (15.5)	1 (0.1)
Michigan	**26,465 (5.2)**	13,069 (5.3)	13,323 (5.2)	133 (0.1)	26,332 (7.7)	11,161 (2.6)	1,059 (1.3)	2,977 (2.1)	4,127 (2.9)	3,067 (3.8)	990 (1.7)	825 (1.5)	234 (0.8)
Minnesota	**55,522 (17.8)**	27,226 (17.8)	28,241 (17.7)	19,113 (19.0)	36,409 (17.2)	50,835 (20.5)	4,313 (6.6)	27,506 (26.6)	15,967 (16.5)	1,935 (18.7)	5,427 (14.7)	2,933 (8.3)	1,380 (4.6)
Mississippi	**3,463 (2.1)**	1,673 (2.1)	1,790 (2.1)	908 (1.7)	2,555 (2.3)	2,113 (2.8)	1,329 (1.5)	—	545 (3.7)	1,336 (2.5)	232 (2.6)	864 (1.7)	465 (1.3)
Missouri	**28,903 (8.7)**	13,991 (8.7)	14,901 (8.8)	9,734 (9.0)	19,169 (8.6)	26,315 (10.5)	921 (1.1)	7,516 (13.3)	15,851 (12.4)	883 (3.2)	2,065 (5.3)	568 (1.5)	353 (0.8)
Montana	**4,655 (8.6)**	2,073 (7.8)	2,198 (7.9)	1,482 (8.7)	3,173 (8.5)	1,804 (9.5)	2,424 (6.9)	—	—	—	1,804 (9.5)	1,477 (9.0)	947 (5.1)
Nebraska	**11,811 (10.2)**	5,780 (10.2)	6,020 (10.1)	3,961 (10.5)	7,850 (10.0)	10,725 (13.7)	1,001 (2.7)	—	—	10,415 (14.7)	310 (4.1)	641 (3.3)	360 (2.0)
Nevada	**6,827 (4.1)**	3,355 (4.1)	3,469 (4.1)	1,949 (3.6)	4,878 (4.3)	6,176 (4.0)	291 (2.1)	4,025 (3.2)	—	2,052 (8.4)	99 (3.5)	270 (2.2)	21 (1.3)
New Hampshire	**7,237 (12.7)**	3,533 (12.6)	3,701 (12.7)	2,311 (12.6)	4,926 (12.7)	4,725 (12.5)	1,922 (9.9)	—	2,877 (15.8)	1,848 (9.5)	—	1,730 (9.7)	192 (12.2)
New Jersey	**48,612 (10.5)**	23,892 (10.5)	24,635 (10.4)	13,993 (9.3)	34,619 (11.1)	48,319 (10.4)	—	15,530 (12.9)	28,292 (9.6)	3,821 (10.7)	676 (5.6)	—	—
New Mexico	**13,520 (12.6)**	6,604 (12.5)	6,794 (12.5)	4,071 (11.8)	9,449 (12.9)	9,471 (13.8)	3,927 (10.2)	—	—	6,780 (15.3)	2,691 (11.0)	3,688 (10.7)	239 (6.1)
New York	**112,570 (11.3)**	55,398 (11.4)	56,923 (11.2)	35,138 (10.6)	77,432 (11.6)	107,622 (11.5)	3,541 (5.8)	67,838 (12.6)	25,646 (9.4)	10,039 (11.6)	4,099 (11.0)	2,763 (6.2)	778 (4.5)
North Carolina	**50,533 (9.2)**	24,506 (9.1)	25,430 (9.1)	15,992 (9.0)	34,541 (9.4)	43,206 (9.8)	3,889 (3.6)	19,336 (15.3)	3,086 (4.1)	18,242 (9.9)	2,542 (4.7)	3,203 (4.0)	686 (2.4)
North Dakota	**4,340 (9.1)**	2,152 (9.3)	2,186 (8.8)	1,456 (9.4)	2,884 (8.9)	2,834 (12.2)	1,275 (5.2)	—	—	—	2,834 (12.2)	505 (3.9)	770 (6.5)
Ohio	**59,516 (9.6)**	28,971 (9.6)	30,345 (9.6)	19,917 (9.9)	39,599 (9.5)	54,742 (11.1)	3,154 (2.6)	28,633 (15.0)	12,944 (10.2)	12,629 (8.3)	536 (2.2)	2,785 (2.8)	369 (1.5)
Oklahoma	**9,336 (4.1)**	4,629 (4.1)	4,705 (4.0)	3,003 (4.1)	6,333 (4.1)	7,596 (4.9)	1,441 (1.9)	3,107 (6.1)	1,793 (5.7)	2,522 (3.9)	174 (2.3)	1,121 (2.5)	320 (1.1)
Oregon	**25,919 (13.0)**	12,648 (13.0)	13,252 (12.9)	9,062 (14.3)	16,857 (12.3)	24,242 (14.5)	1,481 (4.5)	8,025 (22.3)	9,898 (17.1)	3,496 (8.7)	2,823 (8.4)	1,255 (4.5)	226 (4.7)
Pennsylvania	**88,733 (14.3)**	41,546 (13.7)	43,419 (13.6)	29,614 (14.7)	59,119 (14.0)	83,219 (14.9)	1,714 (2.7)	31,081 (21.2)	34,219 (19.8)	14,464 (7.8)	3,455 (6.6)	1,248 (2.6)	466 (2.7)
Rhode Island	**7,562 (15.6)**	3,673 (15.4)	3,879 (15.7)	2,524 (16.2)	5,038 (15.3)	7,055 (14.5)	—	3,811 (11.7)	3,244 (20.1)	—	—	—	—
South Carolina	**14,124 (5.4)**	6,922 (5.4)	7,190 (5.3)	4,370 (5.1)	9,754 (5.5)	12,693 (5.6)	554 (1.6)	—	1,049 (4.8)	11,110 (6.2)	534 (2.0)	378 (1.8)	176 (1.2)
South Dakota	**5,212 (9.6)**	2,527 (9.4)	2,667 (9.6)	1,644 (9.3)	3,568 (9.7)	2,966 (11.0)	2,191 (8.0)	—	—	—	2,966 (11.0)	912 (7.1)	1,279 (8.7)
Tennessee	**18,834 (5.1)**	9,337 (5.2)	9,457 (5.0)	6,401 (5.3)	12,433 (5.0)	17,883 (6.1)	816 (1.1)	8,823 (9.0)	4,119 (5.4)	4,220 (4.7)	721 (2.6)	550 (1.2)	266 (0.8)
Texas	**133,499 (7.5)**	65,793 (7.5)	67,682 (7.5)	38,650 (6.8)	94,849 (7.8)	126,925 (7.9)	2,519 (1.5)	62,698 (7.3)	25,166 (7.5)	36,806 (12.1)	2,255 (2.0)	733 (0.8)	1,786 (2.4)
Utah	**24,148 (11.1)**	11,766 (11.1)	12,341 (11.1)	8,485 (11.9)	15,663 (10.7)	23,034 (11.7)	1,057 (5.2)	12,530 (17.2)	348 (6.8)	8,981 (9.0)	1,175 (6.0)	805 (7.0)	252 (2.8)
Vermont	**8,120 (31.7)**	4,051 (32.2)	4,067 (31.3)	2,859 (34.7)	5,261 (30.3)	3,271 (34.4)	2,862 (17.8)	—	—	—	3,271 (34.4)	1,462 (15.3)	1,400 (21.5)
Virginia	**63,435 (14.0)**	30,851 (13.9)	32,567 (14.0)	19,971 (13.5)	43,464 (14.2)	34,814 (8.5)	549 (1.2)	6,350 (8.8)	25,493 (9.5)	629 (2.0)	2,342 (6.3)	77 (0.7)	472 (1.4)
Washington	**75,287 (18.5)**	37,044 (18.7)	38,120 (18.3)	26,749 (20.5)	48,538 (17.6)	72,066 (19.5)	2,471 (6.7)	39,816 (35.1)	19,645 (15.6)	8,148 (10.3)	4,457 (8.7)	1,992 (6.5)	479 (7.4)
West Virginia	**3,251 (3.9)**	1,570 (3.9)	1,613 (3.8)	962 (3.6)	2,289 (4.1)	2,596 (5.0)	611 (2.0)	—	227 (8.5)	737 (4.5)	1,632 (5.0)	332 (2.4)	279 (1.6)
Wisconsin	**39,543 (13.4)**	19,407 (13.5)	20,100 (13.3)	13,177 (13.8)	26,366 (13.2)	34,279 (15.3)	4,465 (6.4)	7,955 (13.9)	5,731 (12.8)	12,795 (24.9)	7,798 (10.9)	2,594 (7.3)	1,871 (5.4)
Wyoming	**1,233 (4.0)**	617 (4.1)	613 (3.9)	350 (3.7)	883 (4.2)	322 (3.2)	892 (4.3)	—	—	—	322 (3.2)	801 (6.3)	91 (1.2)

* Defined as receipt of ≥1 dose of Pfizer-BioNTech or Moderna COVID-19 vaccine on or after June 20, 2022.

^†^ Persons with state of residence reported as “unknown” (1,258) were not included in jurisdiction-specific counts.

^§^ Persons with sex reported as “unknown” (7,215) were not included in male and female counts.

^¶^ Persons with age reported as zero years at time of vaccination were assumed to be aged ≥6 months.

** Information on the resident's county of residence was not known or was invalid for 88,243 (5.0%) persons.

**FIGURE 1:**
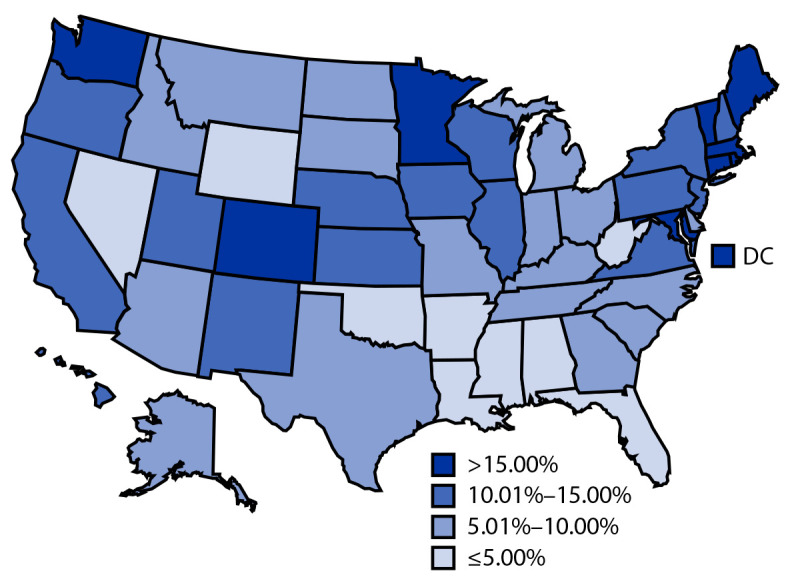
Percentage of children aged 6 months–4 years who received ≥1 dose* of a COVID-19 vaccination series, by jurisdiction — United States, June 20–December 31, 2022 **Abbreviation:** DC = District of Columbia. * Receipt of ≥1 dose of Pfizer-BioNTech or Moderna COVID-19 vaccine on or after June 20, 2022.

Among vaccinated children aged 6 months–4 years, race and ethnicity were known for 71.4%. Among those with known race and ethnicity who received at least the first dose, 7.0% were Black, and 19.9% were Hispanic, whereas these groups account for 13.9% and 25.9%, respectively, of the U.S. population of children aged 6 months–4 years. In contrast, 55.3% of vaccine recipients were White, and 13.4% were Asian children; these groups account for 48.4% and 5.7% of the U.S. population of children aged 6 months–4 years, respectively (*4*) (Figure 2). Race and ethnicity were unknown or not reported for 501,899 (28.6%) children, either because race and ethnicity had not been recorded (24.5%), was reported as “other” (3.6%), or was not reported (0.5%) because of jurisdictional policy or law (Vermont and eight counties in California).

**FIGURE 2:**
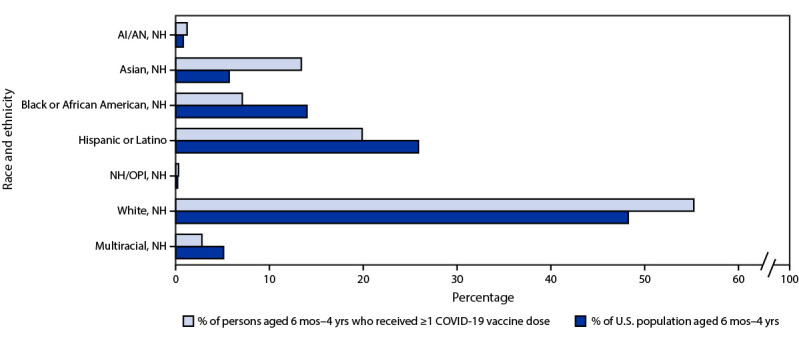
Race and ethnicity* of children aged 6 months–4 years who received ≥1 dose of a COVID-19 vaccination series, by racial and ethnic distribution of the U.S. population^†^ aged 6 months–4 years — United States, June 20–December 31, 2022 **Abbreviations:** AI/AN = American Indian or Alaska Native; NH = non-Hispanic; NH/OPI = Native Hawaiian or other Pacific Islander. * Race and ethnicity was available for 71.4% of persons. ^†^ The U.S. Census Bureau does not include the category “other” as a race category, although immunization information systems in many jurisdictions might report “other.” In this analysis, “other race” was considered unknown, and no comparison with U.S. Census Bureau data was made.

COVID-19 vaccination coverage with ≥1 dose was lower among children aged 6 months–4 years residing in rural counties (3.4%) than among those residing in urban counties (10.5%), according to the two-level urban-rural classification (Table). The six-level classification indicated that coverage was highest (12.5%) among children residing in large metro areas and declined as areas became more rural, with the lowest coverage (2.7%) among children residing in noncore (i.e., most rural) areas. Overall, coverage in 41 jurisdictions was higher in urban counties, in two jurisdictions (Arizona and Wyoming) was higher in rural counties, and in four jurisdictions (Louisiana, Michigan, Mississippi, and Nevada) coverage was similar (i.e., within two percentage points) in urban and rural counties. Coverage comparisons could not be made for four jurisdictions (Delaware, District of Columbia, New Jersey, and Rhode Island) that have only urban counties.

## Discussion

Even after 5 months since COVID-19 vaccines were authorized for children aged 6 months–4 years, coverage with ≥1 dose among this age group substantially lags behind that in older children. Two months after vaccine was approved for children aged 5–11 years and 12–15 years, coverage was 24.0% and 33.3%, respectively, in these age groups (*5*). The low coverage to date in children aged 6 months–4 years is concerning and might indicate challenges to future vaccination coverage, especially given that bivalent booster doses are now authorized for this pediatric population as well.^†††^

Disparities in COVID-19 vaccination coverage that have emerged in the COVID-19 vaccine rollout (*5*) are evident among children aged 6 months–4 years. The lower coverage observed among children residing in rural counties than among those in urban counties is consistent with results from a recent survey which found that 53% of parents of children in rural areas reported that they will “probably or definitely not get their child vaccinated” with COVID-19 vaccines compared with 38% of parents in suburban areas (*7*). However, in Arizona and Wyoming, coverage was higher in rural counties than in urban counties; the reasons for this are not well understood and merit further investigation. Asian and White children were overrepresented among those vaccinated, whereas Black and Hispanic children were underrepresented. Several factors might contribute to these disparities. Black and Hispanic communities have high rates of poverty (19.5% and 17.0% respectively), compared with White communities (8.2%), which might affect parents’ or caregivers’ access to vaccination locations, ability to leave work for or travel to vaccination appointments, or access to primary care providers for pediatric vaccination advice (*8*). In a 2022 Kaiser Family Foundation survey published in July 2022, 47% of parents of children aged 6 months–4 years with household incomes ≥$90,000 reported talking to their pediatrician about a COVID-19 vaccine, compared with only 18% of parents with a household income of $40,000–$90,000 and 28% of parents with a household income of <$40,000 (*9*).

In addition, approximately 40% of Hispanic parents reported that they could not get the vaccine from a place they trust, and approximately one third were concerned about having to pay out-of-pocket for their child to get the vaccine compared with only 13% of White parents (*9*). Approximately 40% of Black parents in that survey reported concern about having to take time off from work to take their child to get vaccinated or to take care of them if they had side effects after receiving the vaccine compared with only 18% of White parents (*9*). Having access to a medical home^§§§^ and a recommendation from a trusted health care provider can help address parental concerns about COVID-19 vaccine safety and effectiveness and can help improve pediatric COVID-19 vaccination coverage (*5*).

Many factors contribute to vaccine hesitancy among parents of the youngest children, ranging from worries about side effects to confusion about information regarding COVID-19 vaccines from federal health agencies (*9*). According to the Kaiser Family Foundation COVID-19 Vaccine Monitor, parental intention to vaccinate children in this age group has remained low, with more than one half the parents of children aged 2–4 years responding in June 2021 that they will “not vaccinate immediately” (*10*) and more than one half of the parents reporting that they will “definitely not” get their children aged 6 months–4 years vaccinated in September 2022.^¶¶¶^ Among parents of unvaccinated children aged 6 months–4 years, approximately 80% were concerned about side effects from the vaccine, and 70% were somewhat or very concerned that the vaccine would not keep their child from getting sick (*9*).

The findings in this report are subject to at least five limitations. First, children who received COVID-19 vaccines from different entities that used different methods for submitting data (e.g., if the first dose was given at a pharmacy and the second dose was given at a mass vaccination site) might not have their first and second doses linked, which could have led to underestimation of the percentage of children who completed the vaccination series. Second, if a child inadvertently received a different recipient identification number when receiving their second dose, first and second doses could not be linked. Third, race and ethnicity were unknown for approximately 30% of children aged 6 months–4 years, which could bias the findings. Fourth, the U.S. Census Bureau does not include “other” as a race category; however, many IIS jurisdictions might report race as “other,” which could affect the interpretation of proportions for this category. Finally, the CDC’s National Center for Health Statistics Urban-Rural Classification was developed in 2013, and counties once classified as rural in 2013 might no longer have been rural in 2022.

An estimated 3 million COVID-19 cases and more than 500 associated deaths have been reported among children aged <5 years since the start of the COVID-19 pandemic (*1*). Children aged 6 months–4 years are now eligible for COVID-19 vaccination; public health practitioners, health care professionals, child care facility and school administrators, and state and local governments can employ evidence-based practices**** to decrease barriers to vaccination and increase confidence in COVID-19 vaccines, which can help reduce COVID-19–associated morbidity and mortality among the nation’s youngest children.

**Summary**

**What is already known about this topic?**

Although severe COVID-19 hospitalization and death occur more commonly among adults, young children are also affected.

**What is added by this report?**

As of December 31, 2022, coverage with ≥1 COVID-19 vaccine dose among young children (those aged 6 months–4 years) was 10.1%, and 5.1% had completed the primary series. Coverage among young children varied by jurisdiction, urbanicity, race, and ethnicity. Five months after the COVID-19 vaccines became available to young children, their vaccination coverage is substantially lower than that in older children.

**What are the implications for public health practice?**

Enhanced evidence-based practices are needed to decrease barriers to vaccination and increase parental COVID-19 vaccine confidence to improve COVID-19 vaccination coverage among young children to reduce associated morbidity and mortality.

## Footnotes

* Defined as having received either ≥1 Pfizer-BioNTech or Moderna vaccine dose.

† Defined as receipt of 2 doses of Moderna or 3 doses of Pfizer-BioNTech COVID-19 vaccines.

§ IISs are confidential, computerized, population-based systems that collect and consolidate vaccination data from providers in 64 public health jurisdictions and can be used to track administered vaccines and measure vaccination coverage. The 64 IIS jurisdictions comprise the 50 U.S. states, eight U.S. territories and freely associated states (Puerto Rico, U.S. Virgin Islands, American Samoa, Northern Mariana Islands, Guam, Marshall Islands, Palau, and the Federated States of Micronesia), and six local jurisdictions (Chicago, Illinois; District of Columbia; Houston, Texas; New York, New York; Philadelphia, Pennsylvania; and San Antonio, Texas).

¶
https://www.cdc.gov/vaccines/covid-19/reporting/vams/program-information.html

**
https://www.cdc.gov/vaccines/covid-19/reporting/overview/IT-systems.html

†† Providers are required to document vaccination in their medical records within 24 hours of administration and to their jurisdiction’s immunization information systems within 72 hours of administration.

§§ Age was calculated based on date of birth provided. However, for nine jurisdictions (Alaska, California, Colorado, Hawaii, Illinois, Massachusetts, North Dakota, Philadelphia, and Virginia) that only report year of birth to CDC, July 1 (i.e., midyear) was used to calculate age, and for five jurisdictions (Arkansas, North Carolina, Oregon, South Carolina, and Vermont) that only report month and year of birth to CDC, the 15th day (i.e., midmonth) was used to calculate age. Persons with age reported as zero years at time of vaccination were assumed to be aged ≥6 months.

¶¶ 45 C.F.R. part 46.102(l)(2), 21 C.F.R. part 56; 42 U.S.C. Sect. 241(d); 5 U.S.C. Sect. 552a; 44 U.S.C. Sect. 3501 et seq.

*** Among persons who received their first dose on or before October 15, 2022, for Pfizer-BioNTech (i.e., ≥11 weeks earlier) or December 3, 2022, for Moderna (i.e., ≥4 weeks earlier). This calculation does not include data from Texas because of data sharing restrictions.

†††
https://www.fda.gov/news-events/press-announcements/coronavirus-covid-19-update-fda-authorizes-updated-bivalent-covid-19-vaccines-children-down-6-months

§§§
https://www.aap.org/en/practice-management/medical-home/medical-home-overview/what-is-medical-home

¶¶¶ https://www.kff.org/coronavirus-covid-19/dashboard/kff-covid-19-vaccine-monitor-dashboard/; https://www.kff.org/coronavirus-covid-19/poll-finding/kff-covid-19-vaccine-monitor-september-2022/

****
https://www.cdc.gov/vaccines/covid-19/planning/children.html
